# Monitoring blast wave evolution and propagation using coupled visual recording and pressure measurements

**DOI:** 10.1038/s41598-026-41174-2

**Published:** 2026-03-19

**Authors:** Sebastian Sławski, Mateusz Polis, Edyta Krzystała, Konrad Szydło, Sławomir Duda, Agnieszka Stolarczyk, Tomasz Jarosz

**Affiliations:** 1 Department of Theoretical and Applied Mechanics, Faculty of Mechanical Engineering, Silesian University of Technology, Konarskiego 18A, 44-100 Gliwice, Poland; 2 Explosive Techniques Research Group, Institute of Industrial Organic Chemistry, Łukasiewicz Research Network, Zawadzkiego 1, 42-693 Krupski Młyn, Poland; 3 Center of Space Technologies, Institute of Aviation, Łukasiewicz Research Network, Al. Krakowska 110/114, 02-256 Warsaw, Poland; 4 Department of Physical Chemistry and Technology of Polymers, Faculty of Chemistry, Silesian University of Technology, Strzody 9, Gliwice, Poland

**Keywords:** Explosive, Combustion, Blast wave, High-speed camera, Pressure, Velocity of detonation

## Abstract

Explosives are a group of special purpose materials, which are important in various branches of industry, such as mining, civil engineering and military. Interest in this type of materials has increased recently, especially due to the current geopolitical situation. Thus, it is important to assess the properties of the explosive as well as evaluate the effects of their use. This paper presents results of research on the analysis of the pressure distribution and characterization of the blast wave produced by two explosives: Ammonal and Heksoflen (95 wt.% RDX / 5 wt.% Viton A). During the research velocity of detonation was measured by four probes placed inside of the prepared charges. The pressure distribution of the blast waves was measured with use of the three pressure probes, placed at various distances from the detonation point. The obtained data were used to determine the explosive constants related to the overpressure, based on which the overpressure prediction was made at various distances for both tested explosives. Moreover, the detonation of the explosives have been recorded with use of Phantom v9.1 high-speed camera. Performed research indicates that, the pressure of the blast wave highly depends on the type of explosive used. Blast wave caused by Heksoflen is characterized by higher maximum pressure and impulse in comparison to Ammonal. After burning of intermediate detonation products differs significantly for the two explosives. After burning of the Heksoflen intermediate products is characterized with wider zone and longer times.

## Introduction

An explosive is a substance which, when suitably triggered, releases a large amount of heat and pressure by way of a very rapid self-sustaining exothermic decomposition reaction^1^. Explosives are a group of special purpose materials, which have been used for many years^2^ and have an important meaning in various branches of industry such as e.g. mining, civil engineering and military^3–6^. Moreover, explosives can be also used during some of the technological processes like welding^7,8^. Explosive are also often used as a propellant^9,10^. Unfortunately, due to explosive properties, they also start to be used as a homemade weapon called improvised explosive device (IED)^11–13^. IED’s are often use to cause a damage to the building, military staff and civilians^14–17^. IED has been the most significant threat by terrorists worldwide and is the leading cause of injury and death for servicemembers operating in Afghanistan and Iraq^17^. Moreover, monthly IED attack frequency during the wars in Iraq and Afghanistan was positively associated with suicide attempts among deployed, previously deployed, and never deployed U.S. Army soldiers, with the overall risk increasing approximately 30% for every 1000 additional IED incidents in a given month^18^.

On the market there are a lot of various explosives which can be classified with use of different criteria, that there exist multiple forms of classification^19,20^. Interest in this type of materials has increased recently in last years, especially due to the current geopolitical situation. A lot of modern application requires the use of explosive and propellant having enhanced performance and reduced sensitivity during its storage and transportation^20^. Thus, there is a need to investigate the properties of new explosive compositions, which are safer and have higher performance compared to nowadays explosive. A lot of facilities around the world are interested in this kind of the research. Chen et al. provides a general strategy to synthesize the NBC-based nanostructured composite energetics. Moreover, the existence of porous cross-linked construction of the prepared explosives has reduced the sensitivities of impact and friction which in turn improved its safety performance^21^. Polis et al. investigated the Ti/WO_3_ nanothermite compositions which exhibit better safety parameters (low friction sensitivity, lack of sensitivity to impact) than other nanothermite formulations containing WO_3_. The electromagnetic irradiation sensitivity and ignition time of the tested samples can be readily controlled by altering the equivalence ratio of the oxidising agent and fuel^22^. Kuterasiński et al. noted that the addition of zeolite Y to ANFO led to the growth of the detonation pressure, temperature, and heat of the explosion^23^. Zygmunt et al. noticed that the porosity of AN prills influences significantly the detonation parameters of ANFO explosives. Higher ANFO mixtures velocity of detonation (VoD) is observed in case of the samples with higher prills porosity^24^. Polis et al. investigated NSTEXs, based on the Ti/CuO nanothermite system supplemented by NTO and noticed that they are characterized by a favorable performances (e.g., a combustion velocity of up to 735 m/s) and high safety parameters^25^. Luo et al. investigated the constant volume combustion properties of Al/Fe_2_O_3_/RDX nanocomposite in an electric detonator. They noticed that the constant volume combustion properties highly depends from the chemical constituents and average size of the particles. Firing energy, ignition delay time and peak pressure decrease gradually when the amount of Al/Fe_2_O_3_ nanothermite increases. The addition of small amount of Al/Fe_2_O_3_ nanothermite to RDX effectively decreases its pressure rise time. Thus, the fast combustion of RDX could be obtained. Improvement in the firing and combustion properties of Al/Fe_2_O_3_/RDX nanocomposite has been noticed when nano-Fe_2_O_3_ with high specific surface area was used as oxidizer, or both cyclohexane and acetone was used as solvent^26^. Paszula et al. analysed how introduction of aluminium–magnesium powder into ANFO charges changed their properties. They noticed that, the introduction of more than 10% of aluminium–magnesium powder resulted in decrease of the VoD. In turn, introduction of less than 10% aluminium–magnesium powder into the tested ammonal mixtures with addition of 3% of flaked aluminium increases the VoD. Moreover, addition of aluminium–magnesium powder into ammonal charges increases the parameters of the air blast wave, increases the glow time of the explosion products cloud by at least 2.5 times and increase the average temperature of the explosion products cloud^27^. Maranda et al. investigated properties of ANFO and ammonal compositions manufactured with use of various type of pours AN, Al (flakes with lateral dimension below 0.075 mm and atomized powder with the grain sizes below 0.15 mm) mixed with diesel oil. Obtained results indicated that regardless of used Al VoD has been increased. However, VoD highly depends on used AN. The highest VoD has been noticed in case of composition with 5% addition of aluminium flakes^28^. Guan et al. investigated RDX composition with addition of PTFE and PA, where the mass fraction of RDX was set at 90%, while that of the polymer powders was 10%. Based on the obtained results Authors noticed that the addition of PA promotes the combustion of RDX, whereas PTFE inhibits the combustion of RDX and alters the composition of its combustion products^29^.

Scientists are interested not only in the properties of explosives but also in their impact on the surrounding area during an explosion. Stanczak et al. investigated a thin-walled aluminum honeycomb structures placed inside of the explosive-driven shock tube. Investigated structures are axially compressed while absorbing the blast energy. It was noticed that the honeycombs of the same height but covering the different areas subjected to compression are characterized by comparable values of the peak and mean equivalent stresses^30^. Baranowski et al. analysed the local blast wave interaction with tire structure. Conducted research showed a possible direction of tire modification which consequently will increase safety of a vehicle^31^. Mazurkiewicz et al. investigated the load carrying capacity of I-column subjected to blast load. Obtained results show that not only the mass of the detonated charge influences the structure behavior. Authors of the paper also noticed that the shape and the initiation point of detonation significantly change the extent of the analysed structure damage^32^. Sielicki et al. analysed the masonry wall behavior under explosive loading^33^. Pyka et al. presents the impact of the used numerical method on the description of the destruction process of a shaft made of EN C45 steel, loaded with a cylindrical TNT explosive charge. Presented imaging of the results provides an information about the destruction process of critical infrastructure elements which are exposed to undesirable threats related to the explosive charges^34^. Olaleye et al. presents a numerical investigation of the blast wave effect on the steel pipe buried beneath the earth^35^. Kciuk et al. present an autonomous system for acquiring measurement data concerning accelerations acting on a test object as a result of impulse, shock, seismic waves, and other impacts. Presented system was used to measure the acceleration of the selected points on an anthropomorphic dummy, which simulates the behavior of the special purpose vehicle crew during the TNT explosion. Presented results demonstrate the efficacy of the anti-explosive seat and floor mat in the context of minimizing the crew’s overload. Moreover, conducted research indicates that use of the tested anti-explosive seat reduced the pelvis acceleration to an acceptable value during the TNT explosion under the vehicle^36^. Klekiel et al. presents the method to analyse a risk of injury of a human body placed inside a vehicle subjected to an explosion^37^. Research concerning on the influence of the blast wave on the human are especially important because of a lot of health issues and traumas occurs after the blast impact^38–40^. Thus, there is a need to analyse both the parameters of explosives and their impact on the environment and the human body.

In most of the works Authors are focused on the measuring of explosive properties as well as explosion products properties, depends on explosive composition and shape. There is a lack of research which connect measured data with image correlation, which allows to observe what is happening during high-energy decomposition step by step. Especially in case of the larger charges. Thus, in the article, the concept of the developed test stand dedicated to measuring the blast wave parameters caused by various explosive has been presented. Developed test stand consisting of pressure sensors, detonation velocity probes as well as high-speed camera is briefly described in Section "Materials and Methods". Developed test stand was used to assess the properties of two types of explosive charges whose preparation process is described in Section "Materials and Methods". Results from the conducted research, as well as the discussion are described in Section "Results and discussion". Moreover, a simple equation has been used to predict overpressure against scaled distance for tested explosives. Article is summarized by main conclusions presented in Section "Conclusions".

## Materials and methods

During the research two types of explosive have been used. First type of the used explosive is Ammonal with chemical composition of: 88.71 wt. % AN, 7.27 wt. % Al and 4.02 wt. % of the paraffin oil. The Ammonal charges were prepared in two step mixing process. At first, the AN grains after drying process (60 °C, 12 h) had been mixed with flaked Al powder in tumble mixer for 15 min, subsequently the calculated amount of paraffin oil was added and the whole system was mixed for next 15 min. After mixing process, the prepared material was placed in constant conditions, in order to facilitate oil permeation into AN pores for 24 h. Second type of the used explosive is Heksoflen—constitute of Class 1 RDX (according to MIL-DTL-398 D standard) with 5 wt. % addition of the Viton A polymer. The material was prepared via wet mixing of RDX with 40% water suspension of Viton A with subsequent drying process (72 h, 60 °C).

Used explosive has been initiated by 16 ± 0.2 g of the A-IX-1 (RDX with wt. 5% addition of the ceresin-stearin) booster placed at the end of the prepared charge. The booster was cylindrical in shape (diameter of 21 mm, height of 30 mm). Moreover, a detonator well (diameter of 8 mm and depth of 18 mm) is located in the axis of the booster. The density of booster was 1.68 g/cm^3^. Boosters were produced by pressing in a steel matrix, under the pressure of 4 MPa. The electrical detonator (Nitrodet 0.2A produced by Nitroerg S.A.) has been placed in the booster detonator well. During the research three samples made of each explosive have been investigated. Information about the charges used during the research are presented in Table 1.

**Table 1:** Charges used during the research.

Designation of the charge	Explosive	Explosive weight (g)	Explosive density (g/cm^3^)	Booster
A1	Ammonal	240.06	0.71	16 ± 0.2 g of the A-IX-1
A2		239.98	0.71	
A3		240.09	0.71	
H1	Heksoflen	321.80	0.95	
H2		323.70	0.96	
H3		322.70	0.95	

**Fig. 1:**
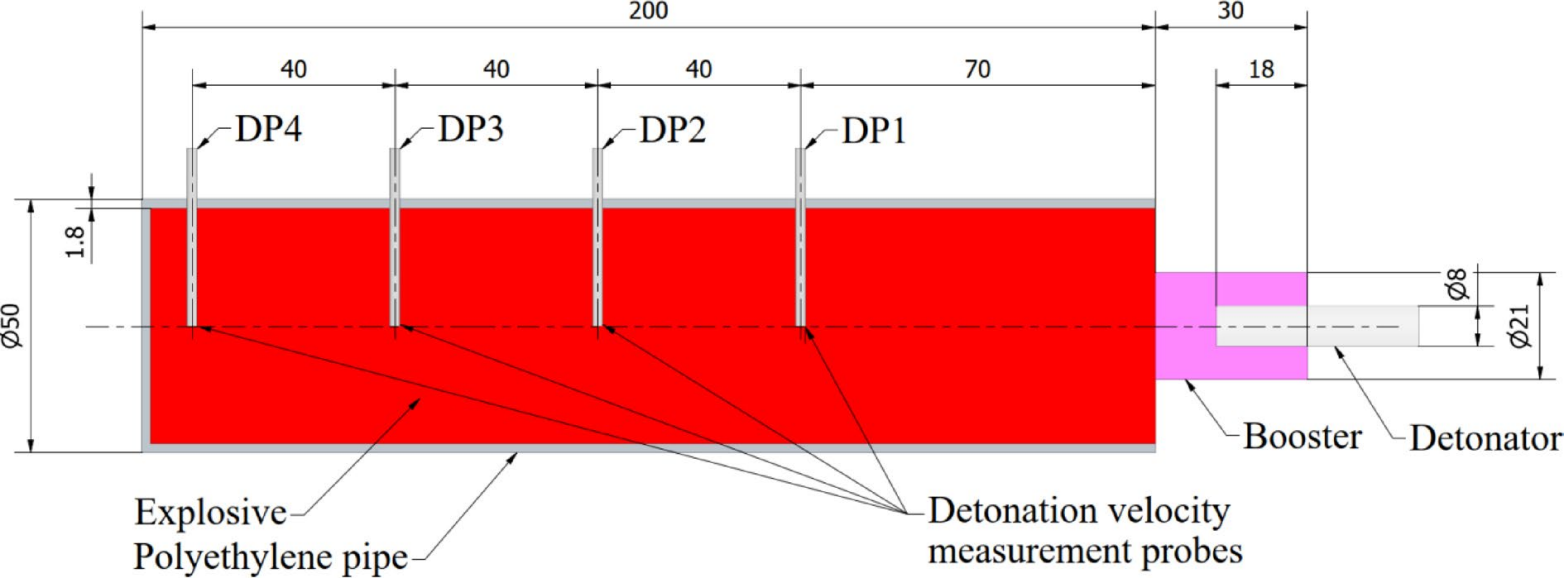
Scheme of the charge used during the research.

Explosive materials has been placed inside of the polyethylene pipes with diameter of 50 mm, wall thickness of 1.8 mm and length of 200 mm. Inside each charge four measuring probes (DP1-DP4) for the velocity of detonation measurement have been also placed. First of the probe was located 70 mm from the contact surface between booster and charge. The subsequent probes were placed inside the charge with maintaining of 40 mm distance between probes. Scheme of the charge used during the research is presented in Fig. 1.

**Fig. 2:**
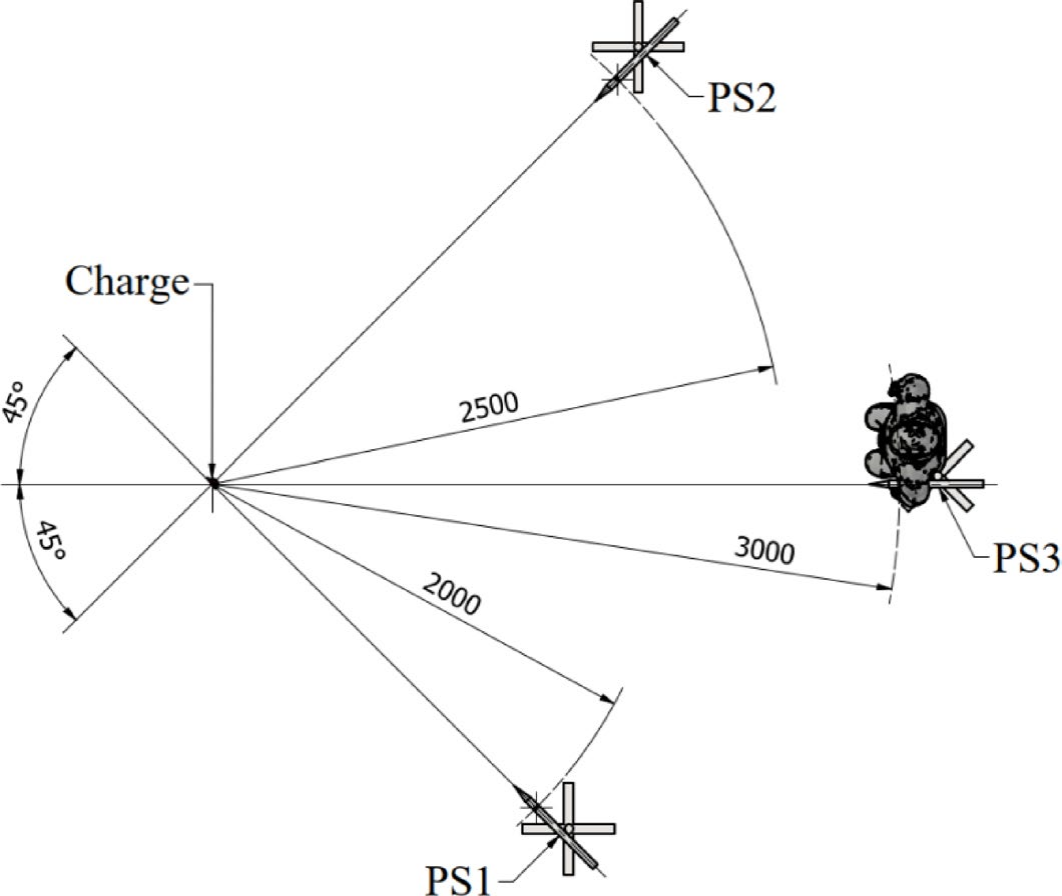
Scheme of the pressure sensors localization.

Charges have been hanged 1 m (measured from the middle of the charge) above the ground level in vertical position (detonator has been turned up). Detonation of the explosive have been recorded with use of Phantom v9.1 (Vision Research, Inc., Wayne, USA) high-speed camera. Camera has been placed approximately 50 m from the explosive. Detonation of each charge has been recorded with sample rate of 2600 FPS. The recording was started directly by a signal from the detonator. Moreover, three blast pressure pencil probes have been used to measure the overpressure of the blast wave at various distances from the detonation point. During the research 137B21B, 137B24B and 137B24B pressure probes (all produced by PCB Piezotronics, INC., Depew, USA) were placed at distances of 2 (PS1), 2.5 (PS2) and 3 m (PS3) from the center of the charge. Sensitivity of used pressure probes was 0.152 mV/kPa, 2.786 mV/kPa and 2.692 mV/kPa respectively. All of the pressure probes were placed 1 m above the ground level (at the same height as a middle point of the investigated charges). Anthropomorphic dummy with weight of 75 kg was also placed near to the PS3. Scheme of the pressure sensors localization is presented in Fig. 2. The overpressure vs time data and velocity of detonation data were collected with sampling of 2 GSa.

## Results and discussion

**Fig. 3:**
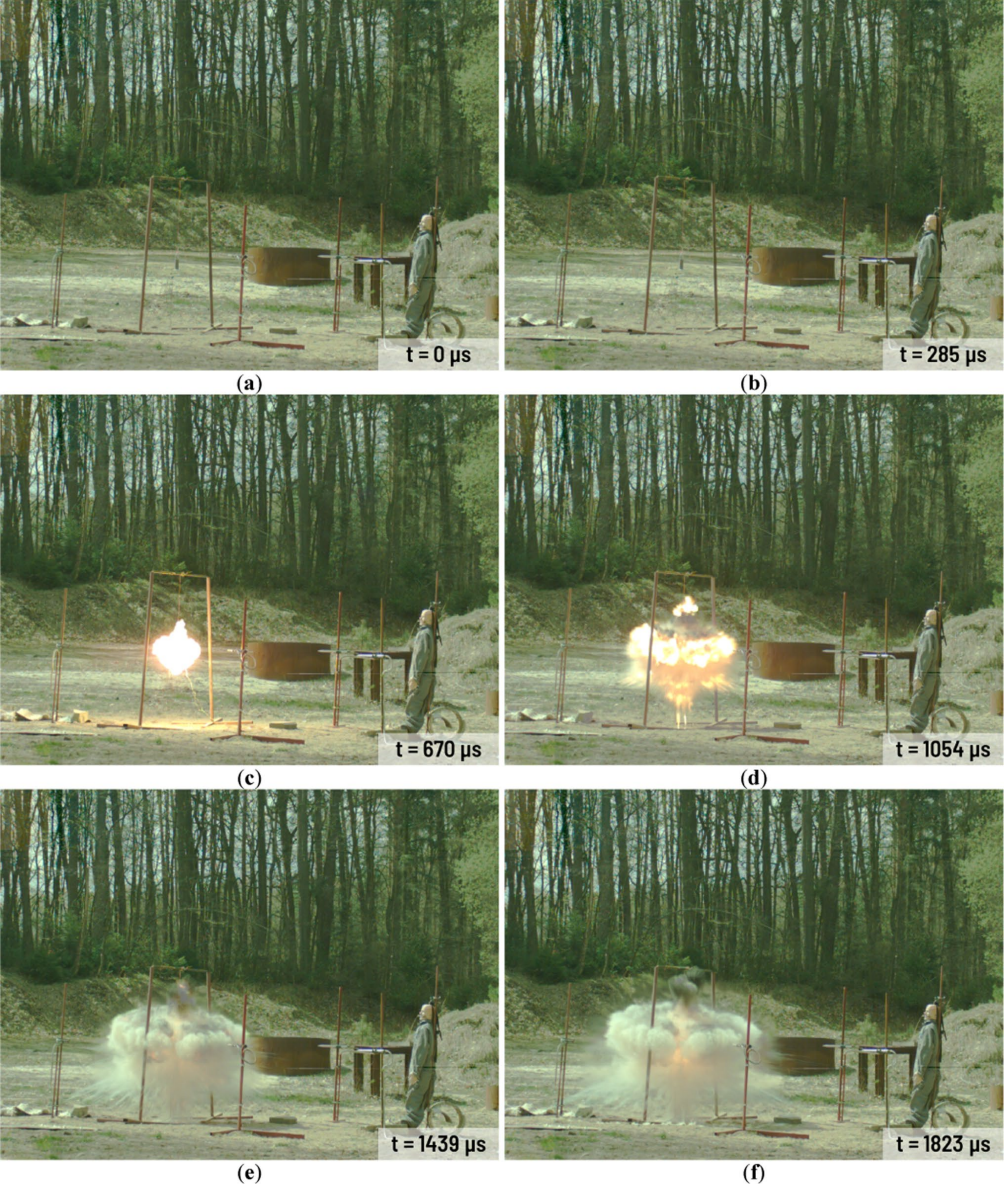

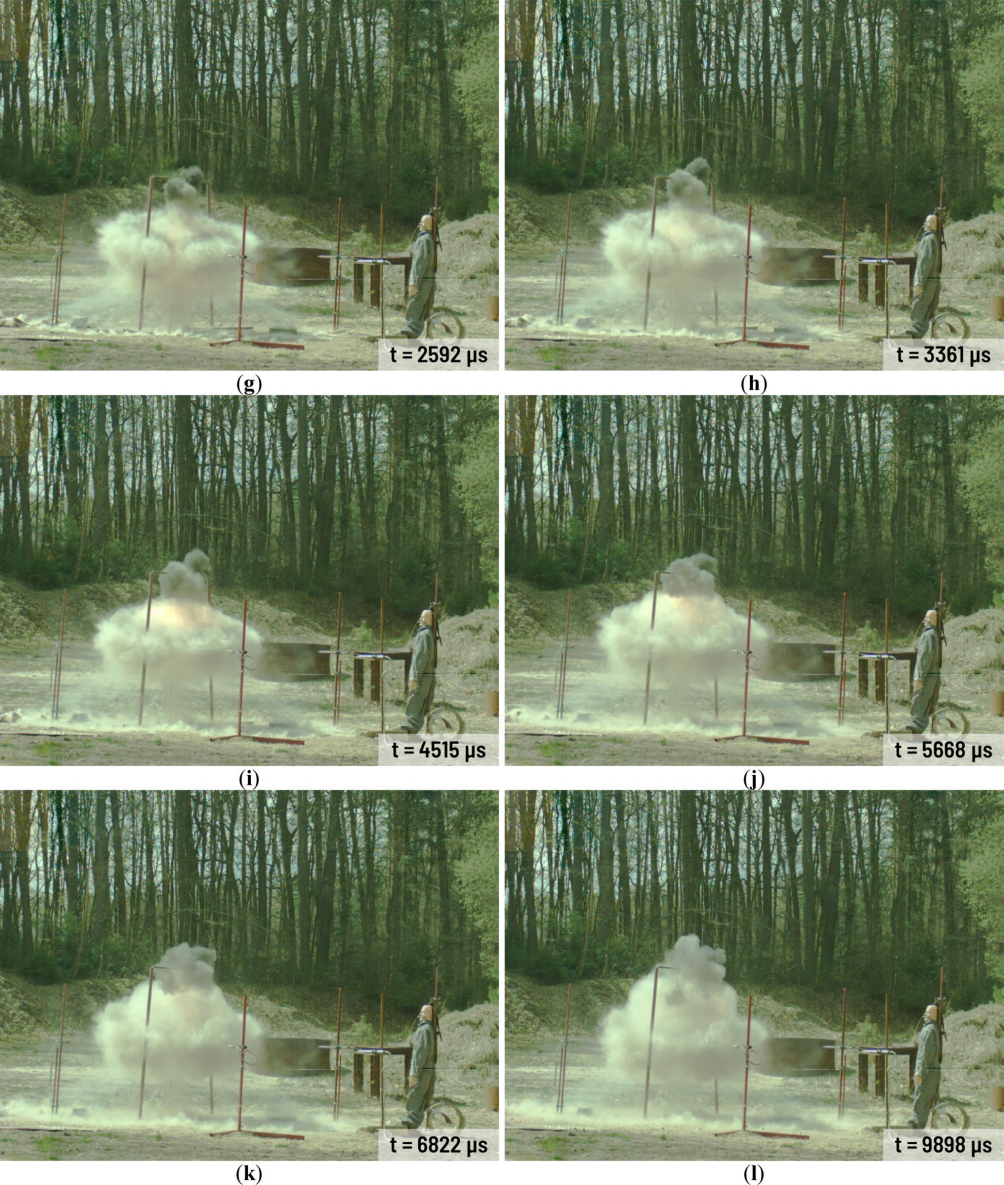
Detonation process of the A3 charge in various time after detonation: (**a**) 0 µs; (**b**) 285 µs; (**c**) 670 µs; (**d**) 1054 µs; (**e**) 1439 µs; (**f**) 1823 µs; (**g**) 2592 µs; (**h**) 3361 µs; (**i**) 4515 µs; (**j**) 5668 µs; (**k**) 6822 µs; (**l**) 9898 µs.

**Fig. 4:**
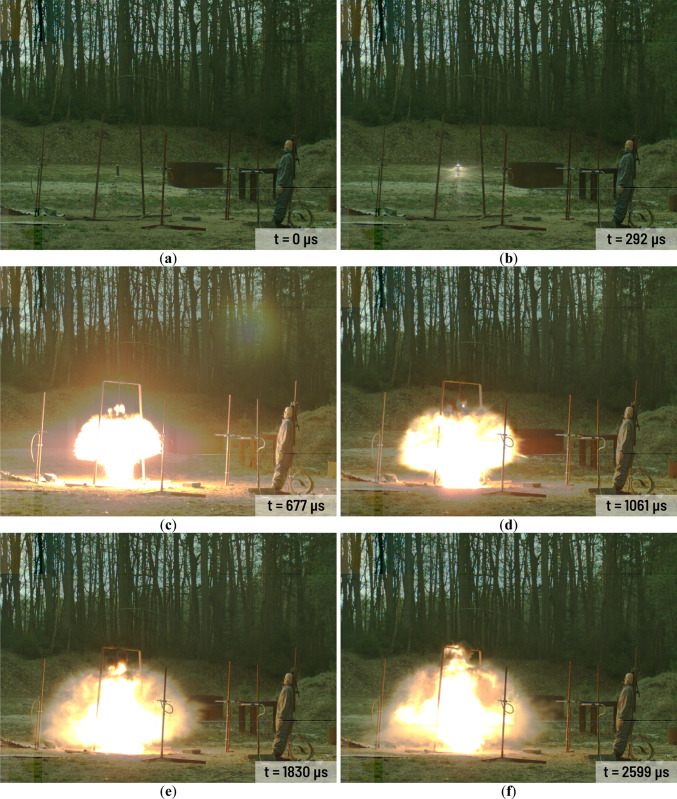

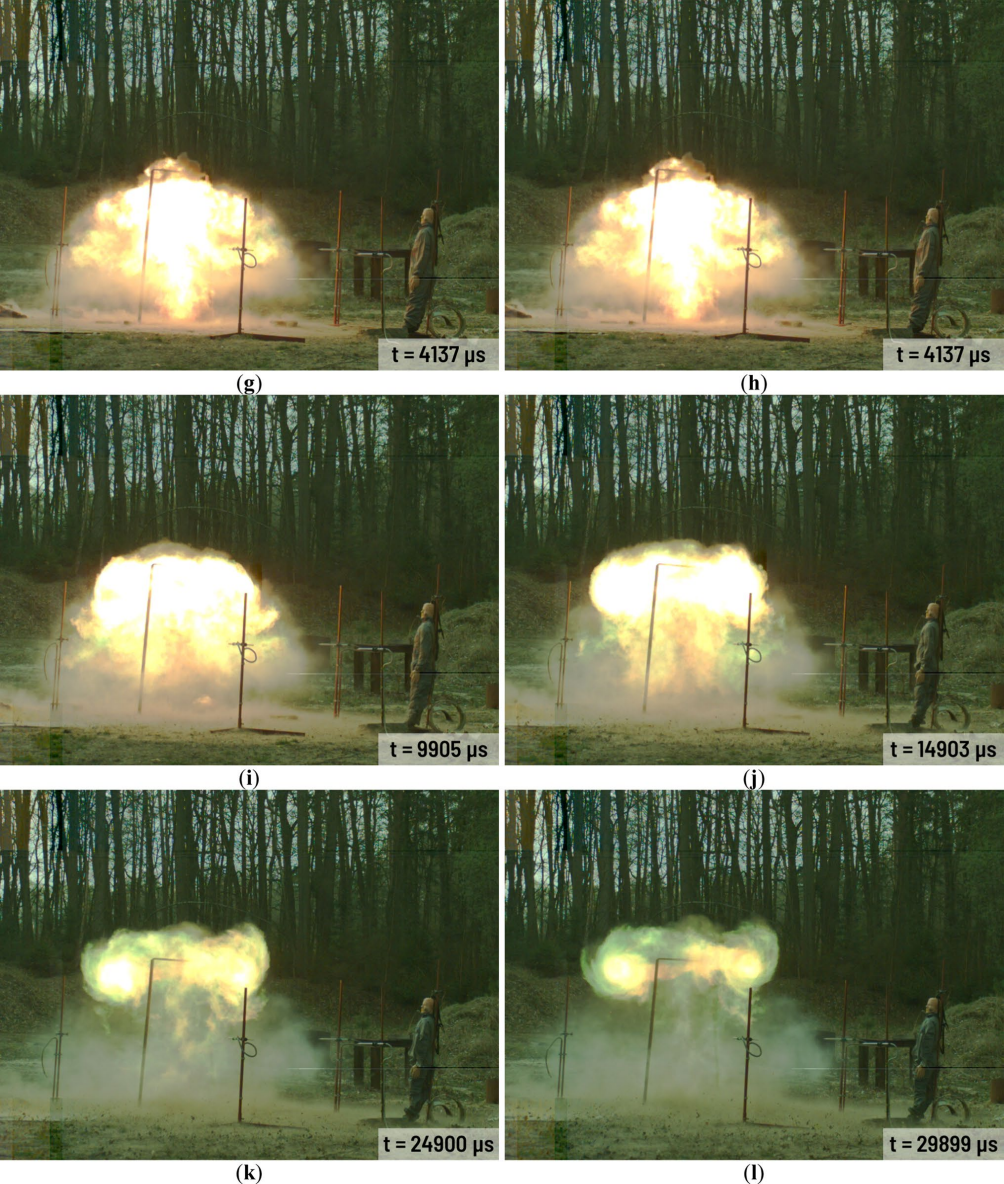
Detonation process of the H2 charge in various time after detonation: (**a**) 0 µs; (**b**) 292 µs; (**c**) 677 µs; (**d**) 1061 µs; (**e**) 1830 µs; (**f**) 2599 µs; (**g**) 4137 µs; (**h**) 6829 µs; (**i**) 9905 µs; (**j**) 14,903 µs; (**k**) 24,900 µs; (**l**) 29,899 µs.

All charges have been investigated during the same day and at stable weather condition. Selected frames from the A3 charge explosion in various time after detonation are presented in Fig. 3. The combustion area in case of A1-A3 charges are rather small (Fig. 3c,d). The gases mixed with solid combustion residues (Al_2_O_3_) and still combusting Al powder are released from the combustion area in short time after the detonation (Fig. 3d). Released gases cover the combustion area and the area close to the charge very quickly (Fig. 3e–l). The high-energy process still occurs near to the point of detonation but it is barely visible because of the released gases and the combustion of the solid residues. Released aerosol remains in the explosion area in the long time after the detonation of each Ammonal charge. Detonation of the H1-H3 charges looks quite different (Fig. 4) in comparison to the A1-A3 charges detonation. The combustion area after the detonation is much larger than in case of the A1-A3 charges (Fig. 4c–f). In case of the A1-A3 charges the combustion area stops to expand approximately 2000 µs after the detonation (Fig. 3f,g). In case of the H1-H3 charges the expansion of the combustion area stops approximately 4100 µs after detonation (Fig. 4g). After this time, the combustion area starts to decrease and starts to float higher and higher above the ground level. At the end of the H1-H3 combustion at least two spiral vortices spinning in the opposite directions are visible (Fig. 4k,l). As time goes by, the vortices get smaller and smaller.

**Fig. 5:**
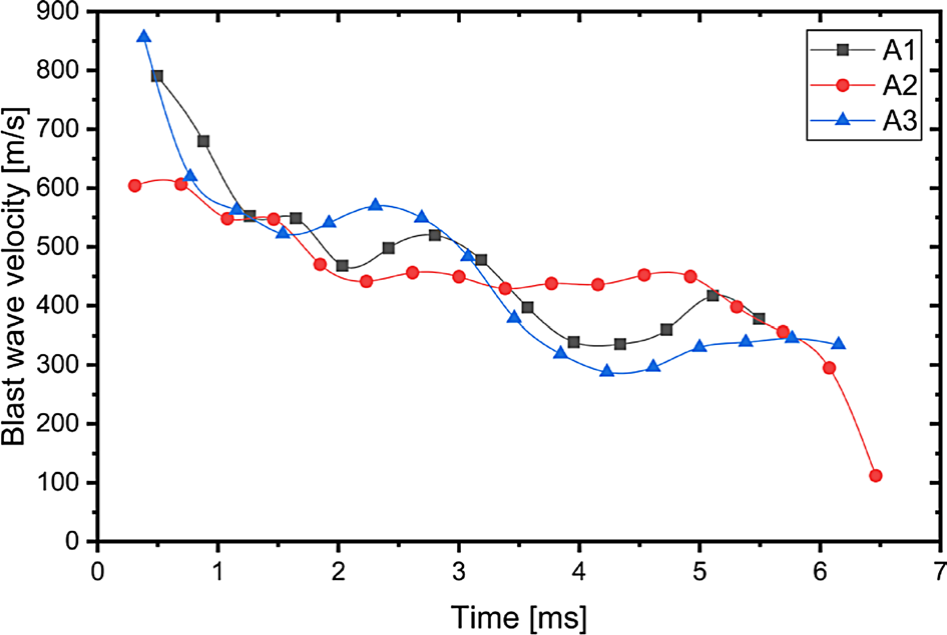
Velocity of the blast wave in case of Ammonal explosive.

**Fig. 6:**
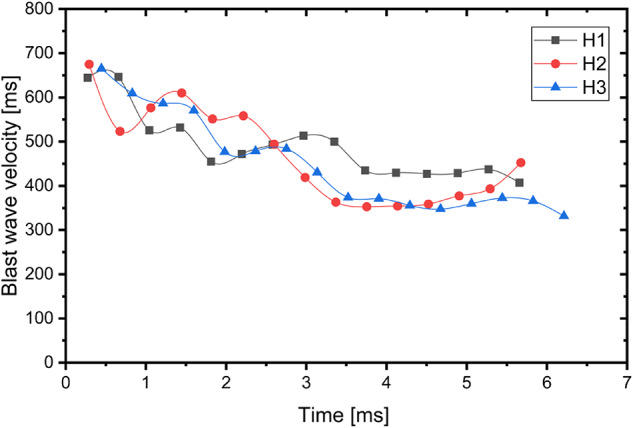
Velocity of the blast wave in case of Heksoflen explosive.

In case of all detonated charges, the blast wave propagating on the surface of the ground and air is well visible. In the case of propagation through the air, the blast wave is visible by the characteristic refraction of the air, which propagated from the detonated charge in all directions. This refraction of the air was used to assess the blast wave propagation velocity based on the recorded videos. The chart of the blast wave propagation velocity in case of the A1-A3 charges is presented in Fig. 5. The chart of the blast wave propagation velocity in case of the H1-H3 charges is presented in Fig. 6. In case of the both type of the investigated explosive the highest blast wave velocity is observed at the beginning (average 750 m/s in case of the Ammonal explosive; average 661 m/s in case of the Heksoflen explosive). Velocity of the blast wave slightly decreases through the time. After approximately 5 ms the average blast wave propagation velocity of the A1-A3 charges is 399 m/s when the average blast wave propagation velocity of the H1-H3 charges is 388 m/s.

The higher values of blast wave propagation of Ammonal charges derivates from presence of Al powder in composition. Even that the amount of gas products is significantly lower in compare with Heksoflen charges, due to post-combustion of Al powder after blast wave front (in expanding gas products) the temperature of gases remains higher for longer period of time. The final measured values are close to velocity of sound in air, and are in line with degradation model of blast wave to a sound wave.

**Fig. 7:**
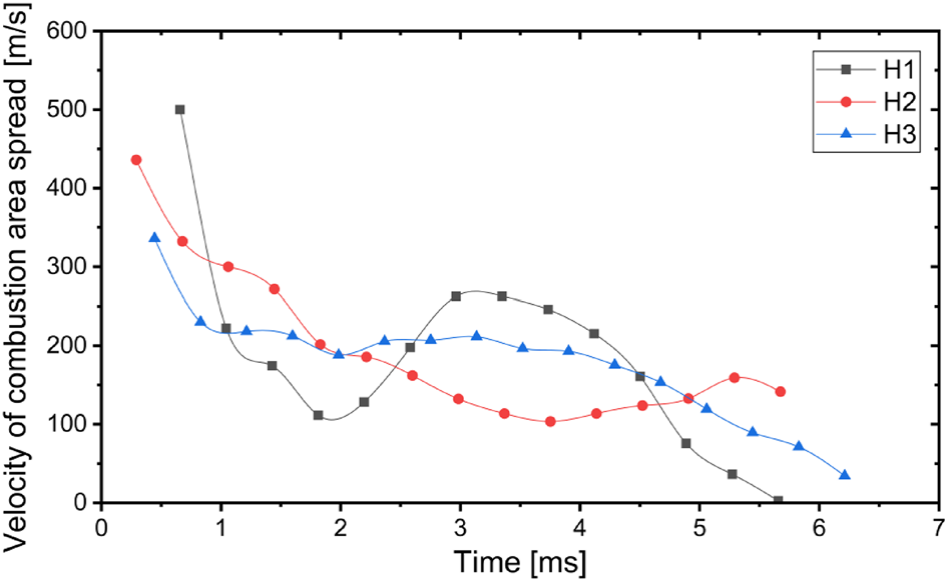
Velocity of the combustion area spread in case of Heksoflen explosive.

In case of the H1-H3 charges the combustion area spread is well visible (Fig. 4). Based on the recorded video, the velocity of this combustion area spread was assessed and presented in Fig. 7. As it could be seen the highest combustion area spread velocity occurs at the beginning of combustion process. The velocity of the combustion area spread decreases very fast in first 2 ms in case of each charge. Next, its starts to decrease in much slighter extent. Depending on the charge, the combustion area spread stops approximately 5–7 ms after detonation. In the case of Ammonal charges combustion area and the area close to the charge is fully covered by released gases approximately 1.5 ms after detonation (Fig. 3). Thus, there was no possibility to measure velocity of the combustion area spread in the case of Ammonal explosive.

Velocity of detonation of all tested charges was measured as a velocity between the subsequent probes (e.g. between first and second) by four probes placed inside the charges (Fig. 1). Table 2 presents the measured velocity of detonation in case of all detonated charges. The average VoD in case of the A1-A3 charges was 2471 m/s when the average VoD in case of the H1-H3 was 6132 m/s. The smallest VoD is observed between the third and fourth measuring probe—between the probes which are located in the highest distance from the detonation point. Thus, the average VoD of the H1-H3 charges is approximately 2.5 higher than in case of the A1-A3 charges. The registered values are in line with typical parameters for used materials, however quite low VoD of H1-H3 charges is related with their low density. The other parameters (e.g. detonation pressure) are strongly related with velocity of detonation, which affects the obtained results.

**Table 2:** Velocity of detonation of the charges.

Charge	Velocity of detonation [m/s] DP_1-2_	DP_2-3_	DP_3-4_	Average
A1	2328	2414*	2414*	2385 ± 50
A2	2849	2368	2568	2595 ± 242
A3	2491	2568	2237	2432 ± 173
H1	6592	6629	5632	6284 ± 565
H2	6968	6629	5934	6510 ± 527
H3	5574	6250	4982	5602 ± 635

*DP_3_ cable connection was damaged during the test, so velocity of detonation was calculated between DP_2_ and DP_4_.

**Fig. 8:**
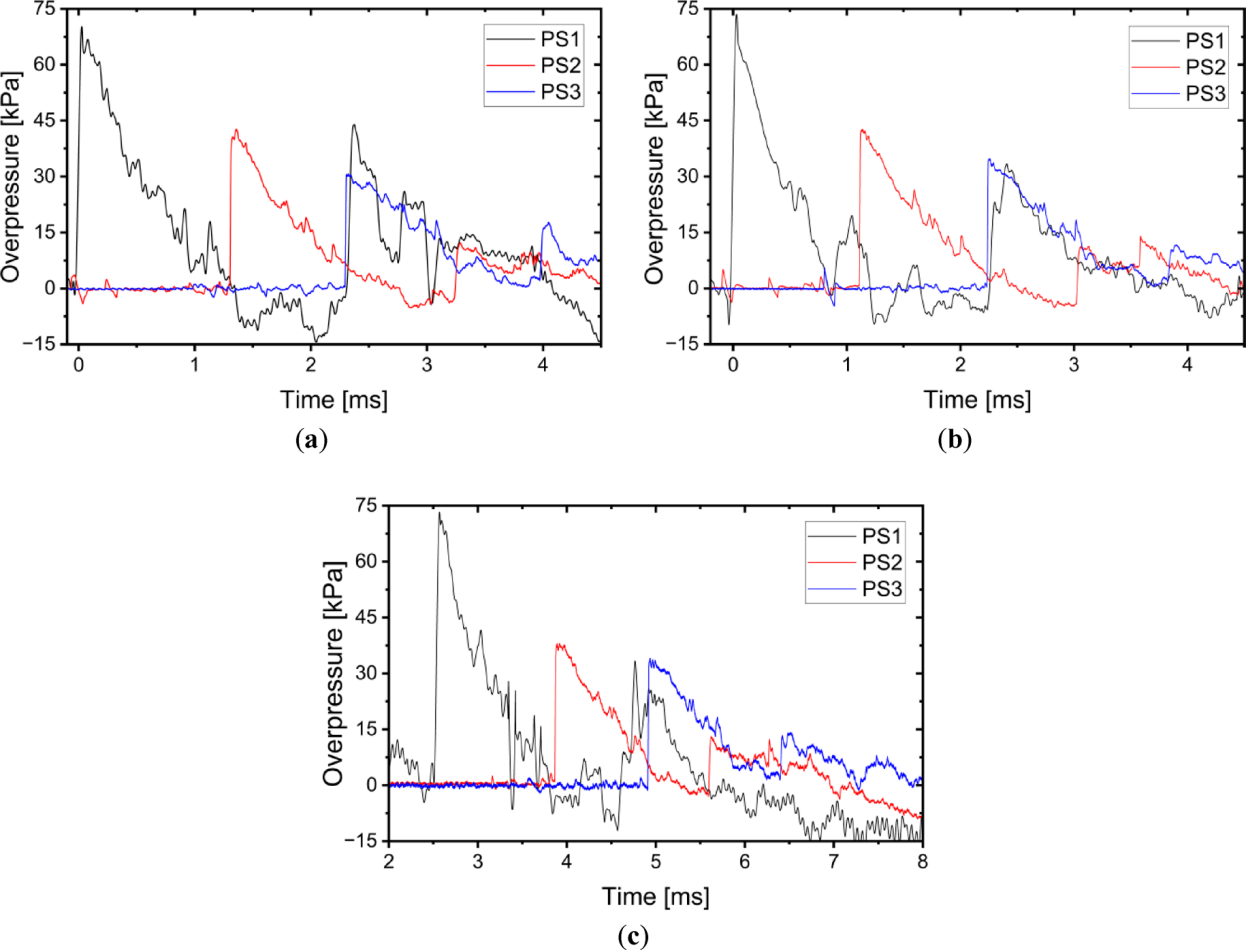
Overpressure generated by the Ammonal explosive measured by pressure sensors in case of: (**a**) A1 charge; (**b**) A2 charge; (**c**) A3 charge.

**Fig. 9:**
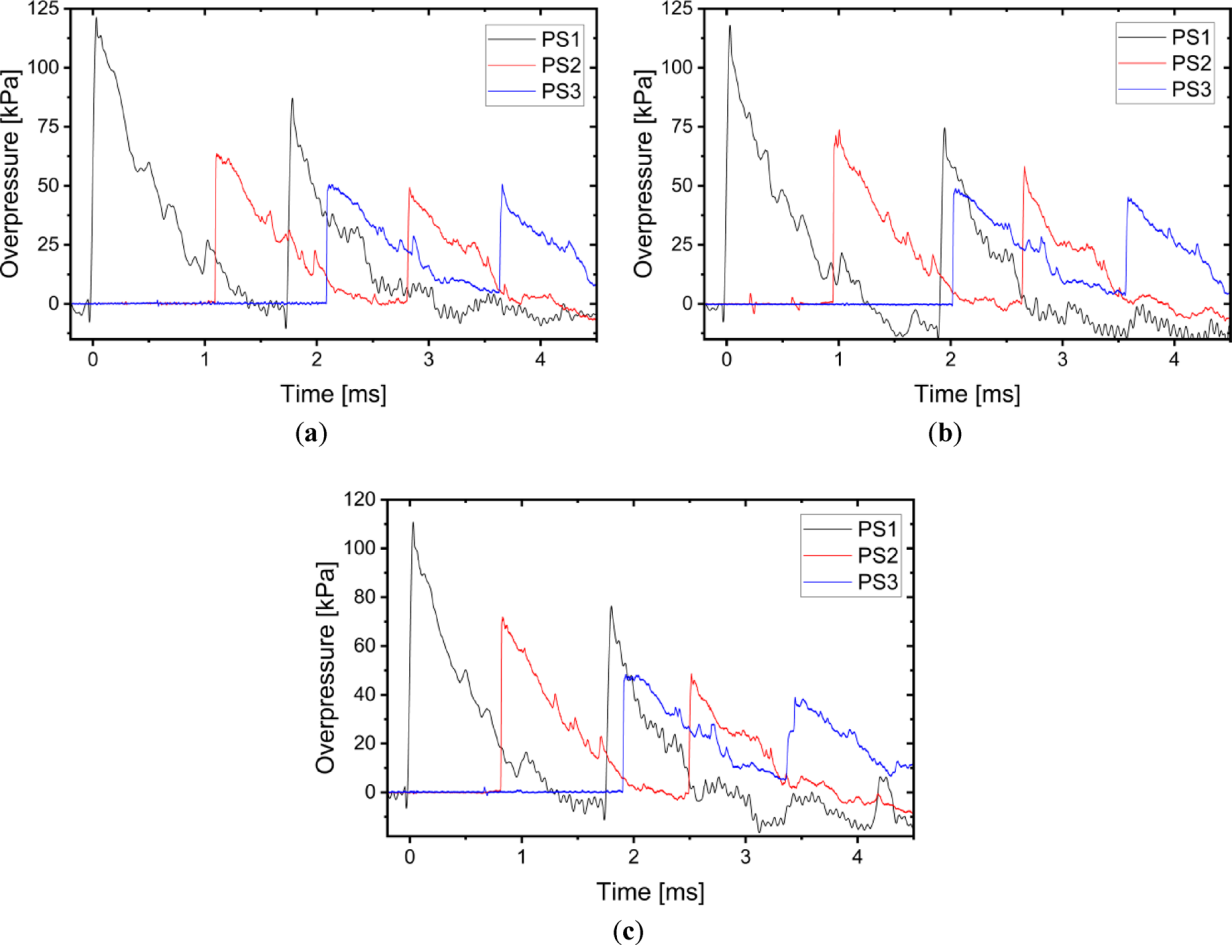
Overpressure generated by the Heksoflen explosive measured by pressure sensors in case of: (**a**) H1 charge; (**b**) H2 charge; (**c**) H3 charge.

During the detonation three pressure sensors were used. Registered overpressures in case of the A1-A3 charges are presented in Fig. 8. Registered overpressures in case of the H1-H3 charges are presented in Fig. 9. In the presented pressure vs time figures the time axis is related with triggering settings of used set-up, namely the initiating of the measurement was trigged by PS1 pressure sensor, not the initiating current pulse on detonator.

Based on the charts presented in Figs. 8 and 9 the parameters such as impulse, maximum overpressure (P_max_) and duration of positive overpressure phase (τ) were identified. Parameters of the blast wave caused by Ammonal explosive are presented in Table 3, when the parameters of the blast wave caused by Heksoflen explosive are presented in Table 4.

**Table 3:** Parameters of the blast wave caused by Ammonal explosive.

Parameter	Sensor	Charge			Average
		A1	A2	A3	
Impulse [Pa*s]	PS1	37.81	32.29	36.93	35.68 ± 2.97
	PS2	22.75	24.00	23.82	23.52 ± 0.68
	PS3	21.72	22.24	22.69	22.22 ± 0.49
P_max_ [kPa]	PS1	78.74	81.37	78.79	79.63 ± 1.50
	PS2	44.06	43.49	41.62	43.06 ± 1.28
	PS3	31.44	35.87	37.04	34.78 ± 2.95
τ [ms]	PS1	1.38	1.23	1.28	1.30 ± 0.08
	PS2	1.51	1.41	1.41	1.44 ± 0.06
	PS3	1.59	1.50	1.48	1.52 ± 0.06

**Table 4:** Parameters of the blast wave caused by Heksoflen explosive.

Parameter	Sensor	Charge			Average
		H1	H2	H3	
Impulse [Pa*s]	PS1	62.48	51.95	50.81	55.08 ± 6.43
	PS2	36.92	37.64	38.28	37.61 ± 0.68
	PS3	34.87*	30.22*	34.93*	33.34 ± 2.70
P_max_ [kPa]	PS1	127.68	119.87	118.56	122.04 ± 4.93
	PS2	64.66	74.38	72.67	70.57 ± 5.19
	PS3	51.36*	49.59*	49.15*	50.03 ± 1.17
τ [ms]	PS1	1.30	1.26	1.33	1.30 ± 0.04
	PS2	1.38	1.44	1.50	1.44 ± 0.06
	PS3	1.62*	1.59*	1.60*	1.60 ± 0.02

*Extrapolated value.

Presented parameters indicated that the impulse and the maximum overpressure measured by all sensors are higher in case of the H1-H3 charges in comparison to the A1-A3 charges. The biggest impulse and overpressure is measured by the sensor located in the shortest distance to the explosive in all analysed charges. The smallest impulse and overpressure is measured by the sensor located in the larger distance to the explosive in all analysed charges. So, the impulse and the maximum overpressure falls down when the distance between the sensors and detonation point increases. This is because the pressure of the blast wave falls down with the increase of the distance between the measuring point and the detonation point. The biggest impulse and maximum overpressure in case of the A1-A3 charges were equal to 35.68 ± 2.97 Pa*s and 79.63 ± 1.50 kPa respectively. The biggest impulse and maximum overpressure in case of the H1-H3 charges were equal to 55.08 ± 6.43 Pa*s and 122.04 ± 4.93 kPa respectively. Thus, the impulse and the P_max_ (measured by PS1) in case of the H1-H3 charges are respectively 1.54 and 1.53 times higher than in case of the A1-A3 charges. Similar proportion (approximately 1.43–1.63) is also visible in the values measured by the other pressure sensors. The maximum overpressure in case of the A1-A3 charges decreases between the PS1–PS2 and PS2–PS3 respectively about 46% and 19%. The maximum overpressure in case of the H1-H3 charges decreases between the PS1–PS2 and PS2–PS3 respectively about 42% and 29%. Therefore, it can be state that the independently from the type of used explosive, the shorter the distance from the detonation point, the faster the pressure drops. The same is observed in case of the impulse.

Knowledge about overpressure distribution at various distances from the detonation point is very important in case of the risk analysis related to the explosives. Such investigation can be done at the test ground but it is very complex—it requires a lot of time and each sample should be investigated in similar conditions what could be problematic in case of long-lasting researches. Therefore, there is a need to use a mathematical methods to predict the overpressure at various distances based on limited experimental data. Shirbhate et al.^41^ and Anas et al.^42^ made a review of existing methods which indicate that a lot of methods require the knowledge about TNT equivalence of tested charge and they are related mainly to describe the over-pressure generated by spherical and hemispherical charges. Held^43^ investigate a cylindrical charges and noticed that the transferred radial momentum is clearly deviating from a spherical detonation wave at near distances and the radial expansion directions of the reaction products are pushed to much higher velocities. Thus, the blast wave parameters distribution obtained for the cylindrical charges are differ in compare to the spherical charges. Knock et al.^44^ used an Eq. (1) to predict the overpressure distribution in case of the cylindrical charges and obtained good correlation between empirical data and calculations, even for simple equation.

1 $$P=K\cdot \frac{M}{{R}^{3}}$$

In Eq. (1) *P* is the peak pressure (kPa), *K* is the constant which depends from the explosive (kPa m^3^ kg^-1^), *M* is the mass of the explosive (kg) and *R* is the distance from the detonation point (m). In presented formula there is no need to calculate the TNT equivalence because of the material constant *K*, what is important because TNT equivalence vary from many factor such as e.g. type of used explosive, explosive density, shape of the charge and from which parameter have been chosen for calculation of TNT equivalent (e.g. heat of detonation, pressure parameters for given distance) etc.

**Fig. 10:**
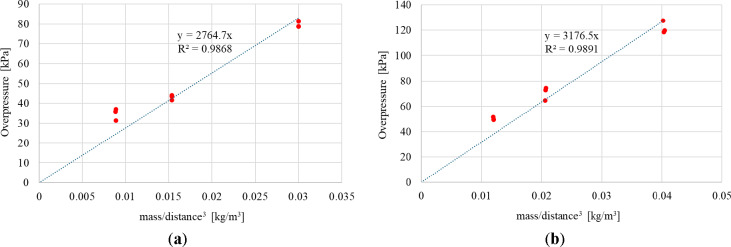
Maximum measured overpressure and its linear regression in case of: (**a**) Ammonal charges explosion; (**b**) Heksoflen charges explosion.

In conducted research material constant *K* have been identified separately for the Ammonal charge and Heksoflen charge based on the experimental data presented in Tables 3 and 4. Measured maximum overpressures as well as its linear regression in case of both investigated explosive are presented against mass/distance^3^ in Fig. 10. The coefficient of determination (R^2^) is 0.9868 in case of Ammonal charge and 0.9891 in case of Heksoflen charge. Material constant *K* have been identified based on line equation as 2765 in case of Ammonal charge and as 3177 in case of Heksoflen charge.

Thus, taking into account the determined constants *K*, Eq. (1) takes the form (2) in case of Ammonal charge and (3) in case of Heksoflen charge.

2 $$P=2765\cdot \frac{M}{{R}^{3}}$$

3 $$P=3177\cdot \frac{M}{{R}^{3}}$$

**Fig. 11:**
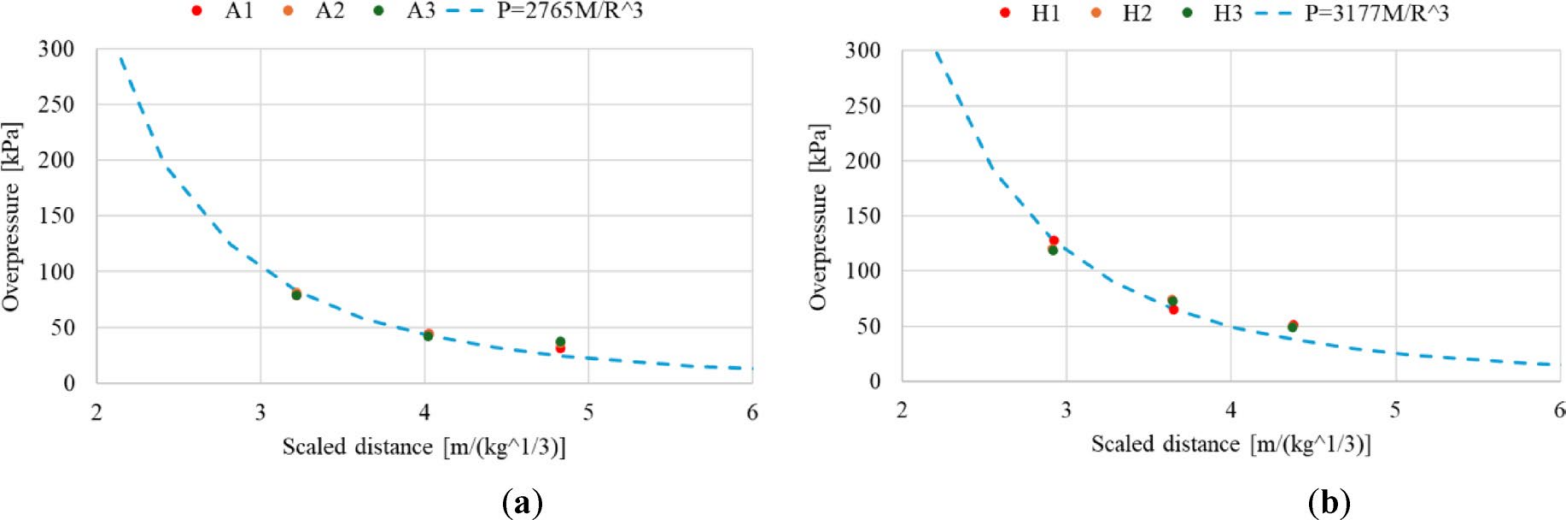
Prediction of overpressure against scaled distance for cylindrical charges compared to experimental data in case of: (**a**) Ammonal charge; (**b**) Heksoflen charge.

Prediction of overpressure for the Ammonal and Heksoflen charges have been calculated based on the Eq. (2) and (3) and presented in Fig. 11 against the scaled distance (*Z*) which is defined based on Eq. (4) ^44^.

4 $$Z=\frac{R}{\sqrt[3]{M}}$$

Based on the experimental data and presented prediction of overpressure it can be stated that the used approach allows to predict the pressure distribution at various distances in good correlation to the experimental data for various cylindrical explosives. In both the case of Ammonal and Hexoflen predicted overpressure is similar to the pressure measured by sensors during the experimental research. Accuracy of the overpressure prediction may be increased with increase of the amount of experimental data, e.g. number of pressure sensors located at various distances from detonation point or by increasing number of tests in similar conditions.

## Conclusions

Conducted research indicate that, the developed test stand allows to measure the properties of the blast wave released by Ammonal and Heksoflen (RDX/Viton A (95/5 wt.%)). Moreover, the use of high-speed camera allows to describe the blast wave propagation and aftercombustion of intermediate products step by step. Data obtained from the recorded videos is an additional supplement to the values measured by the sensors used in the research. Analyse of the videos recorded with a high-speed camera provided valuable information about the combustion process of used explosives. Thus, performed research show how combination of various measuring techniques allows to obtain much more data from the single research. The conclusions from the conducted research are as follows:

- Blast wave properties highly depends from the explosive. Maximum overpressure registered during research is observed in case of Heksoflen charge (1.53 times higher than in case of Ammonal based charge). Blast wave overpressure falls down as the distance from the detonation point increases. The same applies to the impulse which is the highest in case of the Heksoflen based charge (1.54 times bigger in comparison to Ammonal based charge). Impulse also falls down as the distance from the detonation point increases. The dynamics of the maximum overpressure and impulse decline falls down as the distance from the detonation point increases in case of all analysed charges.
- Based on obtained experimental data material constants for Ammonal (K = 2765 kPa m^3^ kg^-1^) and Heksoflen (K = 3177 kPa m^3^ kg^-1^) have been identified. Then, they were used for calculate the relation between overpressure and distance in form of simple equation, which facilitate determination of overpressure values for significantly wider range.
- The combustion zone expansion is significantly different between tested charges. For Ammonal charges, the observed combustion zone is rather small and we may observe the spallation of solid combustion products. At the same time, the post-combustion of Al powder is observed, which confirms Al combustion not only after the CJ plane, but also behind blast wave zone with participation of oxygen from air. This effect strongly contributes on the parameters of blast wave. In case of Heksoflen charges, the combustion zone increase diameter resolutely more and in shorter time. This effect results from higher detonation parameters—as the higher velocity of detonation is connected with higher detonation pressure. Additionally, the amount of gases produced during Heksoflen charges detonation is remarkably higher.

## Data Availability

The data that support the findings of this study are available from the corresponding author, [Sebastian Sławski], upon reasonable request.

## References

[1] Agrawal, J. P. *High Energy Materials: Propellants* (Explosives and Pyrotechnics. WILEY-VCH Verlag GmbH & Co, 2010).

[2] Meyers, S. & Shanley, E. S. Industrial explosives—A brief history of their development and use. *J. Hazard. Mater.* **23**, 183–201. 10.1016/0304-3894(90)85027-Z (1990).

[3] Araos, M. & Onederra, I. Development of a novel mining explosive formulation to eliminate nitrogen oxide fumes. *Min. Technol.* **124**, 16–23. 10.1179/1743286314Y.0000000074 (2015).

[4] Oluwoye, I., Dlugogorski, B. Z., Gore, J., Oskierski, H. C. & Altarawneh, M. Atmospheric emission of NOx from mining explosives: A critical review. *Atmos. Environ.* **167**, 81–96. 10.1016/j.atmosenv.2017.08.006 (2017).

[5] Khomenko, O., Kononenko, M., Myronova, I. & Savchenko, M. Application of the emulsion explosives in the tunnels construction. *E3S Web Conf.* **123**, 01039 (2019). 10.1051/e3sconf/201912301039

[6] Yan, S. W. & Chu, J. A new explosive method for soil improvement. *Proc. Inst. Civ. Eng. Geotech. Eng.* **157**, 259–265. 10.1680/geng.2004.157.4.259 (2004).

[7] Findik, F. Recent developments in explosive welding. *Mater. Des.* **32**, 1081–1093. 10.1016/j.matdes.2010.10.017 (2011).

[8] Vigueras, D. J., de Renero, C. T. & Inal, O. T. Explosive and impact welding: Technical review. *Mater. Technol.* **22**, 200–204. 10.1179/175355507X236740 (2007).

[9] Pradhan, S. K., Kedia, V. & Kour, P. Review on different materials and their characterization as rocket propellant. *Mater. Today Proc.* **33**, 5269–5272. 10.1016/j.matpr.2020.02.960 (2020).

[10] Koch, E.-C. Insensitive high explosives: V. Ballistic properties and vulnerability of nitroguanidine based propellants. *Propellants Explos. Pyrotech.* **46**, 174–206. 10.1002/prep.202000220 (2021).

[11] Gill, P., Horgan, J. & Lovelace, J. Improvised explosive device: The problem of definition. *Stud. Confl. Terror.* **34**, 732–748. 10.1080/1057610X.2011.594946 (2011).

[12] Horváth, T. & Ember, I. Characteristics of homemade explosive materials and the possibilities of their identification. *L. Forces Acad. Rev.* **26**, 100–107. 10.2478/raft-2021-0015 (2021).

[13] András, D. Evolution” of improvised explosive devices (IED) in the light of technical development. *Műszaki Katonai Közlöny* **32**, 49–61. 10.32562/mkk.2022.1.4 (2022).

[14] Grant, M. & Stewart, M. G. Probabilistic risk assessment for improvised explosive device attacks that cause significant building damage. *J. Perform. Constr. Facil.* **29**, B4014009. 10.1061/(asce)cf.1943-5509.0000694 (2015).

[15] Miller, A. et al. Injuries from civilian under-vehicle improvised explosive devices: An analysis of the Israeli National Trauma Registry during the years 2006–2020. *Eur. J. Trauma Emerg. Surg.* **48**, 3813–3819. 10.1007/s00068-021-01739-4 (2022). 34175970 10.1007/s00068-021-01739-4

[16] Maitland, L. et al. Analysis of 983 civilian blast and ballistic casualties and the generation of a template of injury burden: An observational study. *eClinicalMedicine*10.1016/j.eclinm.2022.101676 (2022). 36204004 10.1016/j.eclinm.2022.101676 PMC9530474

[17] Kang, D. G., Lehman, R. A. & Carragee, E. J. Wartime spine injuries: Understanding the improvised explosive device and biophysics of blast trauma. *Spine J.* **12**, 849–857. 10.1016/j.spinee.2011.11.014 (2012). 22197184 10.1016/j.spinee.2011.11.014

[18] Ursano, R. J. et al. Frequency of improvised explosive devices and suicide attempts in the U.S. army. *Mil. Med.* **182**, e1697–e1704. 10.7205/MILMED-D-16-00270 (2017). 28290945 10.7205/MILMED-D-16-00270 PMC5459305

[19] Zapata, F. & García-Ruiz, C. Chemical classification of explosives. *Crit. Rev. Anal. Chem.* **51**, 656–673. 10.1080/10408347.2020.1760783 (2021). 32397736 10.1080/10408347.2020.1760783

[20] Anniyappan, M., Talawar, M. B., Sinha, R. K. & Murthy, K. P. S. Review on advanced energetic materials for insensitive munition formulations. *Combust. Explos. Shock Waves* **56**, 495–519. 10.1134/S0010508220050019 (2020).

[21] Chen, L. et al. Nitrated bacterial cellulose-based energetic nanocomposites as propellants and explosives for military applications. *ACS Appl. Nano Mater.* **4**, 1906–1915. 10.1021/acsanm.0c03263 (2021).

[22] Polis, M. et al. Ti/WO_3_, a nanothermite for special purposes: An experimental study. *Def. Technol.* **41**, 1–12. 10.1016/j.dt.2024.06.010 (2024).

[23] Kuterasiński, Ł et al. Variously prepared Zeolite Y as a modifier of ANFO. *Materials* **15**, 5855. 10.3390/ma15175855 (2022). 36079238 10.3390/ma15175855 PMC9457274

[24] Zygmunt, B. & Buczkowski, D. Influence of ammonium nitrate prills’ properties on detonation velocity of ANFO. *Propellants Explos. Pyrotech.* **32**, 411–414. 10.1002/prep.200700045 (2007).

[25] Polis, M. et al. Novel NSTEX system based on Ti/CuO/NC nanothermite doped with NTO. *Energies* **17**, 3675. 10.3390/en17153675 (2024).

[26] Luo, Q., Liu, G., Zhu, M. & Jiang, X. Constant volume combustion properties of Al/Fe_2_O_3_/RDX nanocomposite: The effects of its particle size and chemical constituents. *Combust. Flame* **238**, 111938. 10.1016/j.combustflame.2021.111938 (2022).

[27] Paszula, J., Maranda, A., Kukfisz, B. & Putko, P. Investigation of the explosive characteristics of ammonium nitrate and aluminium-magnesium alloy powder mixtures. *Energies* **15**, 8803. 10.3390/en15238803 (2022).

[28] Maranda, A., Papliński, A. & Gałęzowski, D. Investigation on detonation and thermochemical parameters of aluminized ANFO. *J. Energ. Mater.* **21**, 1–13. 10.1080/07370650305585.2 (2003).

[29] Guan, S., Wang, Z., Yin, J., Hao, R. & Ji, Q. Effect of polymer compositions on the combustion of RDX explosive. *Chem. Eng. J.* **527**, 171775. 10.1016/j.cej.2025.171775 (2026).

[30] Stanczak, M., Fras, T., Blanc, L., Pawlowski, P. & Rusinek, A. Blast-induced compression of a thin-walled aluminum honeycomb structure—experiment and modeling. *Metals* **9**, 1350. 10.3390/met9121350 (2019).

[31] Baranowski, P., Małachowski, J. & Mazurkiewicz, Ł. Local blast wave interaction with tire structure. *Def. Technol.* **16**, 520–529. 10.1016/j.dt.2019.07.021 (2020).

[32] Mazurkiewicz, L., Malachowski, J. & Baranowski, P. Blast loading influence on load carrying capacity of I-column. *Eng. Struct.* **104**, 107–115. 10.1016/j.engstruct.2015.09.025 (2015).

[33] Sielicki, P. W. & Łodygowski, T. Masonry wall behaviour under explosive loading. *Eng. Fail. Anal.* **104**, 274–291. 10.1016/j.engfailanal.2019.05.030 (2019).

[34] Pyka, D. et al. Analysis of pulse load of a steel roller in the numerical simulation method BT—Fatigue and fracture of materials and structures. In *Fatigue and Fracture of Materials and Structures: Contributions from ICMFM XX and KKMP2021* (eds Lesiuk, G. et al.) 39–45 (Springer International Publishing, 2022). 10.1007/978-3-030-97822-8_5.

[35] Olaleye, K., Pyka, D., Bocian, M. & Jamroziak, K. Numerical modelling of an impact resistance analysis of EN C45 steel pipe buried beneath the ground surface loaded with a detonation wave generated by an explosion of a cubic TNT charge. *J. Phys. Conf. Ser.* **2647**, 82006. 10.1088/1742-6596/2647/8/082006 (2024).

[36] Kciuk, S., Krzystała, E., Mężyk, A. & Szmidt, P. The application of microelectromechanical systems (MEMS) accelerometers to the assessment of blast threat to armored vehicle crew. *Sensors* **22**, 316. 10.3390/s22010316 (2022). 10.3390/s22010316 PMC8749562 35009858

[37] Klekiel, T., Sławiński, G., Świerczewski, M., Bogusz, P. & Będziński, R. Risk of injury in lumbar spine during explosion of low-mass charge under vehicle. *AIP Conf. Proc.* **2078**, 20078. 10.1063/1.5092081 (2019).

[38] Wightman, J. M. & Gladish, S. L. Explosions and blast injuries. *Ann. Emerg. Med.* **37**, 664–678. 10.1067/mem.2001.114906 (2001). 11385339 10.1067/mem.2001.114906

[39] Zhang, J. K. et al. Blast-related traumatic brain injuries secondary to thermobaric explosives: Implications for the War in Ukraine. *World Neurosurg.* **167**, 176-183.e4. 10.1016/j.wneu.2022.08.073 (2022). 36028113 10.1016/j.wneu.2022.08.073

[40] Taylor, P. A., Ludwigsen, J. S. & Ford, C. C. Investigation of blast-induced traumatic brain injury. *Brain Inj.* **28**, 879–895. 10.3109/02699052.2014.888478 (2014). 24766453 10.3109/02699052.2014.888478 PMC4046872

[41] Shirbhate, P. A. & Goel, M. D. A critical review of blast wave parameters and approaches for blast load mitigation. *Arch. Comput. Methods Eng.* **28**, 1713–1730. 10.1007/s11831-020-09436-y (2021).

[42] Anas, S. M. & Alam, M. Comparison of existing empirical equations for blast peak positive overpressure from spherical free air and hemispherical surface bursts. *Iran. J. Sci. Technol. Trans. Civ. Eng.* **46**, 965–984. 10.1007/s40996-021-00718-4 (2022).

[43] Held, M. Impulse method for the blast contour of cylindrical high explosive charges. *Propellants Explos. Pyrotech.* **24**, 17–26. 10.1002/(SICI)1521-4087(199902)24:1<17::AID-PREP17>3.0.CO;2-D (1999).

[44] Knock, C. & Davies, N. Predicting the peak pressure from the curved surface of detonating cylindrical charges. *Propellants Explos. Pyrotech.* **36**, 203–209. 10.1002/prep.201000001 (2011).

